# Migraine through puberty and menopausal transition—data from the population-based Norwegian Women and Health study (NOWAC)

**DOI:** 10.1186/s10194-025-02083-3

**Published:** 2025-06-20

**Authors:** Nora Stensland Bugge, Kjersti Grøtta Vetvik, Karl Bjørnar Alstadhaug, Tonje Braaten

**Affiliations:** 1 Department of Community Medicine, UiT The Arctic University of Norway, Tromsø, Norway; 2 Department of Neurology, Akershus University Hospital, Lørenskog, Norway; 3 NorHead– Norwegian Centre for Headache Research, Norwegian University of Science and Technology, Trondheim, Norway; 4 Department of Neurology, Nordland Hospital, Bodø, Norway; 5 Department of Clinical Medicine, UiT The Arctic University of Norway, Tromsø, Norway

**Keywords:** Migraine, Menopause, Menarche, Postmenopausal women, Norwegian Women and Health study

## Abstract

**Background and purpose:**

Migraine considerably affects women during their reproductive years. This cross-sectional study uses data from the Norwegian Women and Health study (NOWAC) and investigates the typical age at migraine onset and cessation in women and assesses how reproductive milestones affect migraine patterns.

**Methods:**

4825 women with a history of migraine were included in the study. Participants completed a questionnaire that procured detailed information on their migraine characteristics and reproductive histories.

**Results:**

Average ages at migraine onset and cessation were 27.8 and 49.7 years, respectively. Migraine onset after age 50 was reported in 9.2% of the participants. Although 80.7% reported cessation before age 60, 46.3% continued to experience migraines postmenopause. Women with migraine with aura were more likely to report migraine onset before menarche than those with migraine without aura.

**Conclusion:**

Migraines usually resolve during the fifth decade of a woman’s life and menstruation cessation does not necessarily equate to migraine cessation, as almost half of the women continued to experience migraines postmenopause, and one in five after 60 years. Migraine symptom persistence in a significant proportion of postmenopausal women underscores the need for continued management and research on the factors influencing migraine prevalence in later life stages.

**Graphical Abstract:**

Age at last migraine attack by migraine type. Orange dotted line represents mean age at menopause.

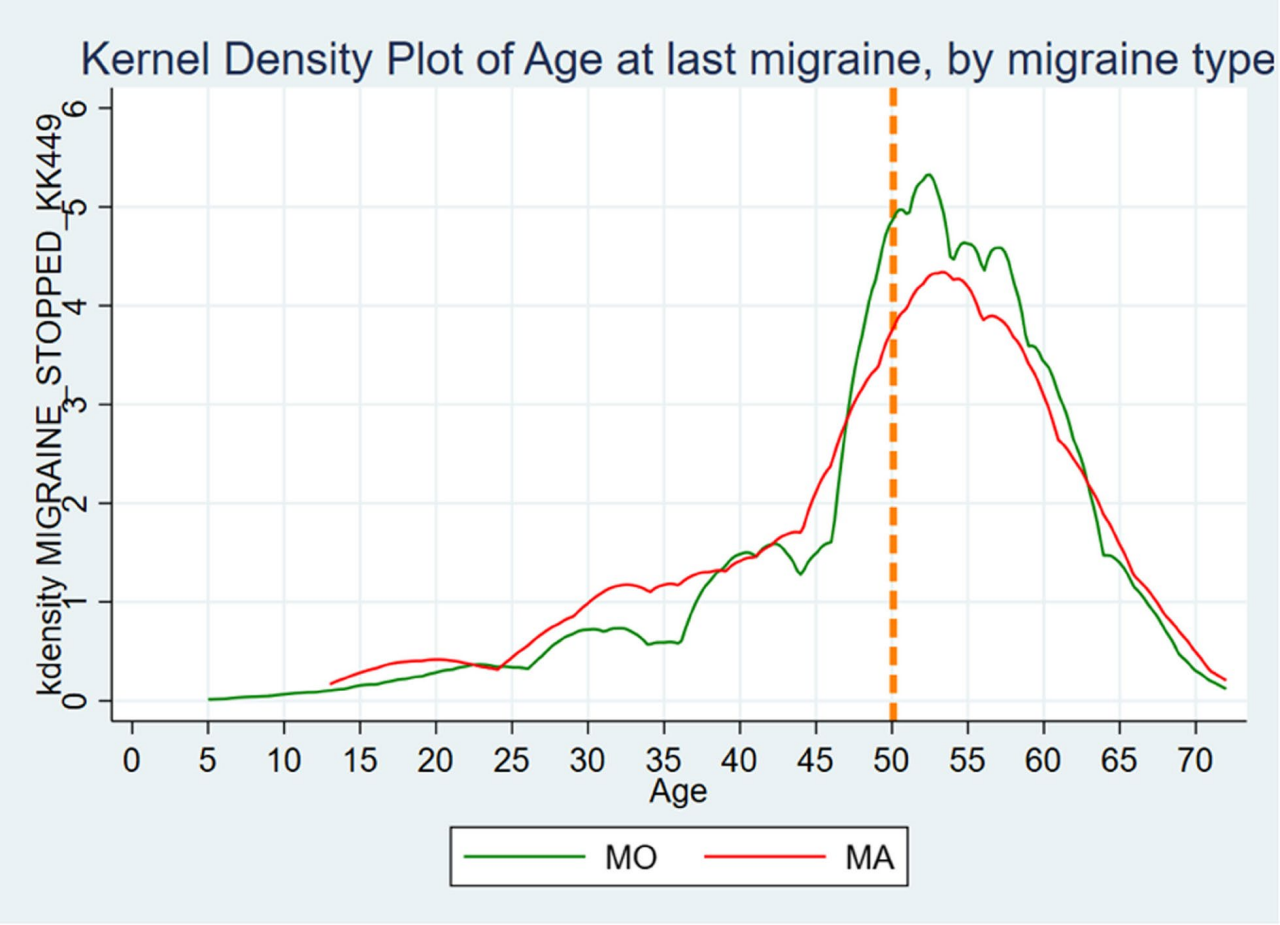

**Supplementary Information:**

The online version contains supplementary material available at 10.1186/s10194-025-02083-3.

## Background

Migraine is a highly prevalent primary headache disorder that occurs in 17.0% of women and 8.6% of men each year [1]. Sex plays a crucial role, and migraine usually affects women during their reproductive years. Epidemiological studies show that female hormones influence the incidence and features of migraine attacks in women [2]. This link is evident in the menstrual-related timing of migraine attacks [3], a decrease in migraine prevalence during late pregnancy [4], and a further decrease after menopause [5–7]. Frequent and erratic fluctuations in hormonal levels are correlated with migraine exacerbation in several women during the perimenopausal period [8]. Up to one-third of women with migraine experiences migraine aura (MA), and it is somewhat more common in men compared to women. Migraine without aura (MO) primarily drives the burden of total migraine disease and is carried by women [9]. Migraine attacks directly linked to menstruation are typically without aura [10].

Migraine’s life course in women, particularly the age at which migraine usually resolves, as well as its occurrence later in life and in postmenopausal women, is unclear [11–13]. The prevailing view, supported by literature, is that migraine symptoms decrease with age, especially following menopause [5–7]. Nonetheless, reports show that approximately 10% of women > 65 years old continue to experience active migraine [1]. Furthermore, the migraine prevalence in older populations is increasing, which correlates with the global demographic aging trends [14]. Migraine presentation in older adults may vary from that in the younger population, with relatively increased reports of MA, aura without migraine headache, and aura with non-migraine headaches [15, 16].

This study aims to identify the age at onset and cessation of migraine in women and study its relationship with menarche and menopause.

## Materials and methods

### Study population

The present study utilized data from the Norwegian Women and Health Study (NOWAC), an ongoing population-based health survey initiated in 1991, to investigate the factors influencing cancer development and survival among Norwegian women. Additionally, the study included self-reported information on other health outcomes, including migraine, obtained through questionnaires. The entire NOWAC study includes 172 478 Norwegian women born between 1927 and 1965, and the study has been described in detail elsewhere [17].

To ensure adequate representativeness in estimating absolute and relative risk, women invited to participate in NOWAC were randomly selected from Norway’s national population register [18]. The overall response rate in NOWAC was 52.7%. Women who answered the baseline questionnaire were sent follow-up questionnaires within 4–13 years (response rate: 80% for the second and 79% for the third questionnaire). A total of 23,223 (75%) women returned a fourth questionnaire containing detailed questions about migraine, described in further detail later, which made them eligible for inclusion in the present study. The fourth questionnaire was sent to the participants in 2017, and the participants were all between 60 and 74 years old. To explore the pattern of onset and cessation of migraine this study analyzed data exclusively from the fourth questionnaire, resulting in a cross-sectional study design due to the single timepoint of data collection.

**Fig. 1:**
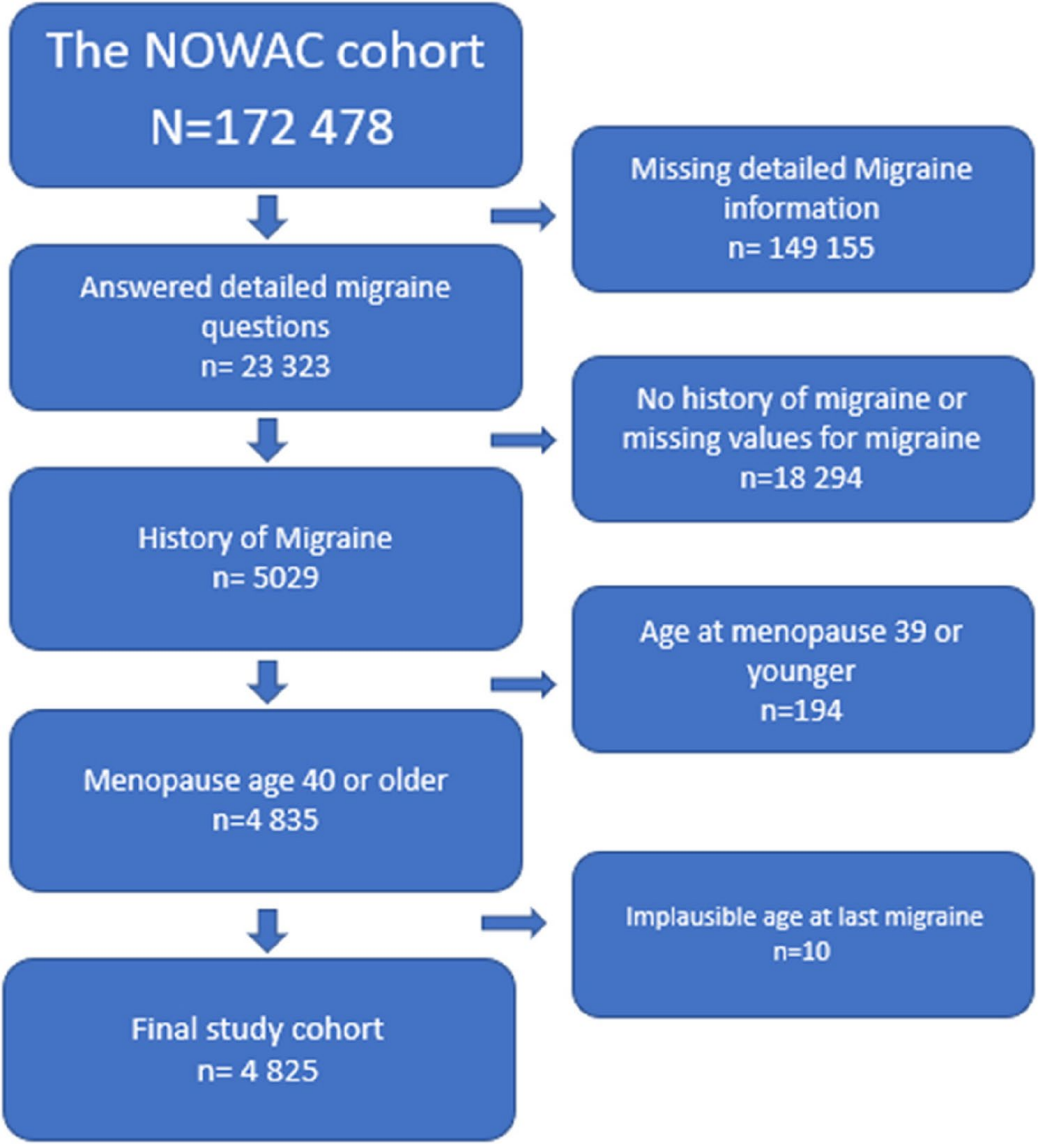
Definition of the cohort

Of these 23,223 women, 5029 reported a history of migraine. This study excluded a subset of 194 women (3.8%) who reported menopause at age ≤ 39. Consequently, the eligible study population comprised 4835 women. Among them, 10 women reported implausible values for age at the last migraine episode. The remaining 4825 women constituted the final study population (Fig. 1).

Regarding missing information; In this study, 332 (10.8%) women without data on age at last migraine or confirmed active migraine in the past 12 months were excluded from the analysis related to the age at migraine cessation. Additionally, 556 women (11.5%) could not be included in the analysis of age at migraine onset because of missing information on when their migraine began. Furthermore, 52 (1.1%) and 162 (3.4%) women were excluded from the analyses of migraine onset relative to menarche and cessation relative to menopause, respectively, due to missing menarche and menopause ages. Among the 162 women excluded for missing menopause data, some might still have been menstruating, as they were 60–74 years old at the time of their last questionnaire.

### The questionnaires

The questionnaire with detailed migraine questions was sent to the participants in 2017. The questions covered several topics, including menarche, menopause, and migraine. A set of questions based on the International Classification of Headache Disorders (ICHD-3) for migraine [19] was included in the fourth questionnaire, which included questions regarding age at first and last migraine and headache characteristics. Participants who responded ‘Yes’ to the question ‘Have you ever had migraine? Yes/No’ were categorized to the migraine group. Those with missing responses to this question or who initially answered ‘No’ but subsequently provided detailed information about their migraine headaches (e.g. headache characteristics, typical duration, and intensity) were reassigned to the migraine group. A validation study from 2020 evaluated the agreement between validated migraine questions, based on the International Headache Society’s diagnostic criteria, and the simple question ‘Have you ever had migraine?’ in a subcohort of the NOWAC. The study reported a kappa value of 0.71, indicating good consistency (unpublished results, Bugge NS and Braaten T). This suggests that the question ‘Have you ever had migraine?’ is an acceptable method of identifying women with migraine.

The participants were asked if they had visual disturbances (aura) before onset of headache, and whether this happened “Never/Rarely”, “Sometimes” or “Most of the time”, the women who reported “Most of the time” were assigned to the MA group. The women who reported to “Never/Rarely” or “Sometimes” was assigned to the MO group.

Self-reported age at first and last menstruation was used to determine age at menarche and age at menopause. Equally, self-reported age at first and last migraine attack defined age at onset and cessation of migraine.

Migraine onset at menarche was defined when the age at migraine onset matched the age at menarche. Similarly, migraine cessation at menopause was defined when the age at menopause matched the age at last migraine. No migraine after menopause was defined when the age at menopause was equal to or less than the age at last migraine. Migraine after menopause was defined when the age at menopause was less than the age at last migraine.

### Statistical analysis

The aims of the statistical analysis were to investigate the age at which the first and last migraine appeared, and the number of years before and/or after menopause the women experienced migraine.

Characteristics of the study sample were presented as percentage distributions with means with standard deviations (SD) for continuous variables, and percentage distribution for categorical variables.

The Mann–Whitney U and Chi-square tests were used for ordinal and nominal variables, respectively, to examine the presence of a crude difference in women with MO compared to those with MA.

Univariate kernel density estimates were created to show the age at migraine onset and cessation for all women with migraine and those with MO and MA.

Statistical analyses were performed using STATA version 17. The significance level was set to 5%. The reported *p* values are not corrected for multiple testing, as we considered the magnitude of the differences in women with MO compared to MA rather than the statistical significance.

### Ethics

All participants provided written informed consent. The NOWAC study has been licensed by the Norwegian Data Inspectorate (2002/2241) and the present study is approved by the Regional Committee for Medical and Health Research Ethics (REC-635219).

## Results

The women in this study were 34–49 years old at baseline and 60–74 years old when they returned the fourth and last questionnaire, containing detailed migraine questions. Furthermore, their mean age was 66.3 (standard deviation (SD) = 4.2) years old.

### Migraine prevalence

Of the 20,255 women eligible to be included in the study, 23.8% (*n* = 4825) reported ever having experienced migraine.

**Fig. 2:**
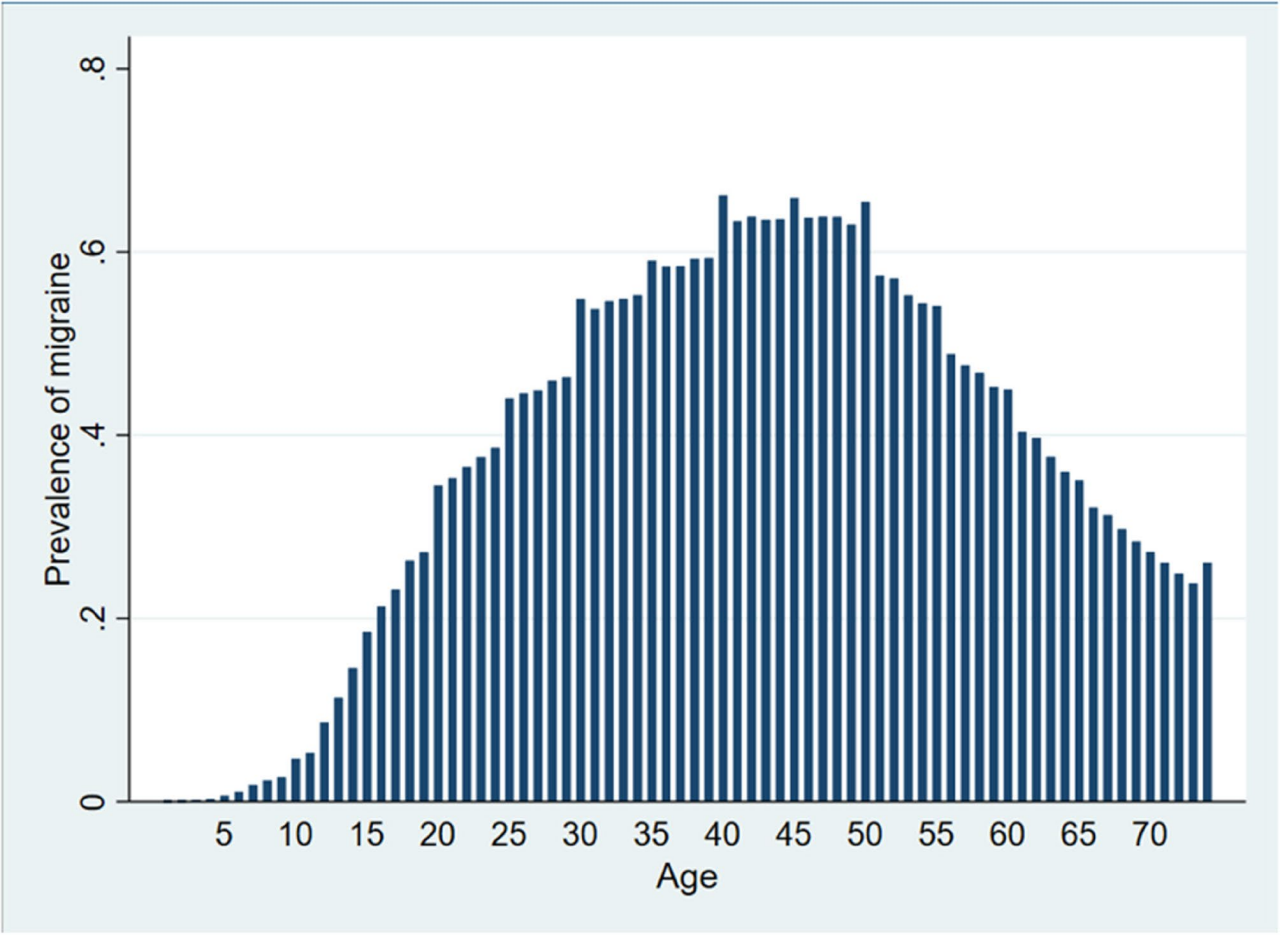
Age-specific prevalence of migraine

Figure 2 shows the age-specific prevalence of migraine in the study population. The prevalence demonstrates a clear age-related pattern, increasing steadily from early adulthood, peaking between the ages of 30 and 50, and subsequently declining with advancing age. The highest prevalence is observed in individuals aged approximately 40 to 45 years. This distribution suggests a significant age-related variation in migraine occurrence, with the condition being most common during middle adulthood.

### Age at first and last migraine

Mean age at migraine onset was 27.8 years (SD = 13.3), with a tendency toward lower age among women with MA compared to women with MO (26.7 vs. 27.7 years, *p* = 0.01).

**Fig. 3:**
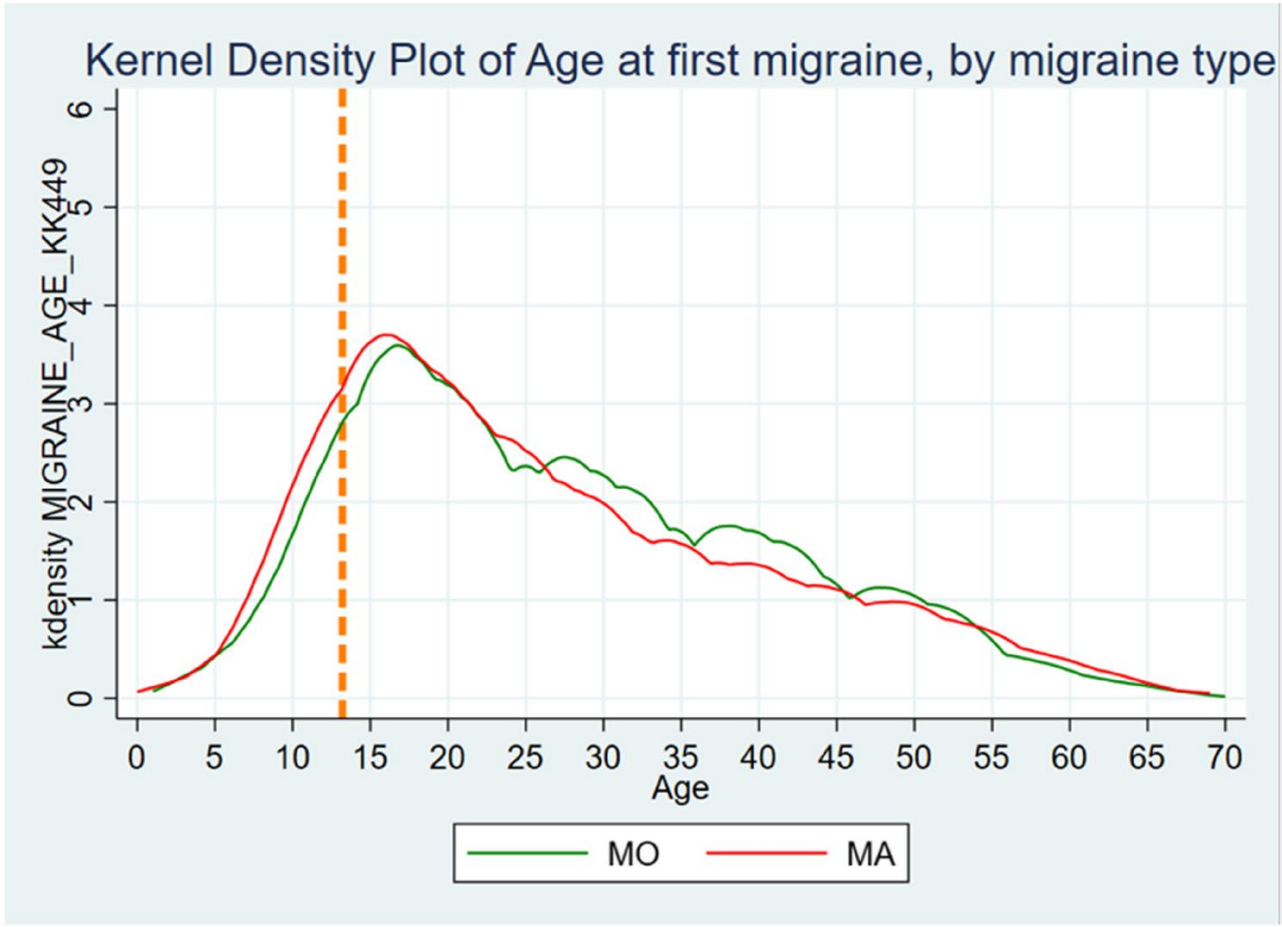
Age at first migraine attack by migraine type. Orange dotted line represents mean age at menarche

**Fig. 4:**
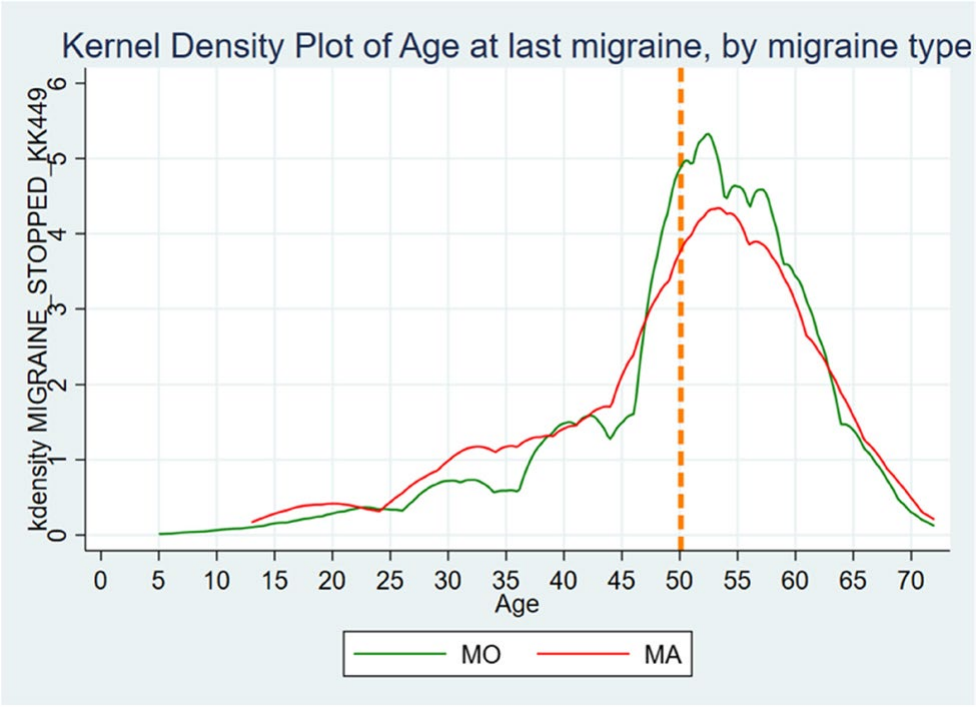
Age at last migraine attack by migraine type. Orange dotted line represents mean age at menopause

Mean age for migraine cessation was 49.7 (SD = 11.3) years, with no substantial difference between the migraine subtypes (*p* = 0.087). Table 1; Figs. 3 and 4 summarize these findings.

**Table 1:** Migraine

	**All women with migraine, No. (%)**	**Migraine without Aura, No. (%)**	**Migraine with Aura, No. (%)**	***P*-value**
**Lifetime prevalence** **Ever had Migraine**				
Yes	4825 (23.8)	2270 (65.9)	1177 (34.1)	
No	15430 (76.2)			
Missing	2360			
**Age at first Migraine**				
< 10	127 (3.0)			
10–19	1227 (28.7)			
20–29	1040 (24.4)			
30–39	846 (19.8)			
40–49	636 (14.9)			
50–59	325 (7.6)			
60+	68 (1.6)			
Mean (SD)	27.8 (13.3)	27.7 (13.3)	26.7 (13.8)	0.01*
Missing	566			
**Age at last Migraine**				
< 10	3 (0.1)			
10–19	51 (1.9)			
20–29	114 (4.2)			
30–39	225 (8.3)			
40–49	513 (18.9)			
50–59	1284 (47.3)			
60–69	499 (18.4)			
70+	26 (0.9)			
Mean (SD)	49.7 (11.3)	50.7 (10.6)	49.5 (11.7)	0.087*
Missing	322			

* = Mann-Whitney U-test

Notably, 393 (9.2%) of the women reported the onset of migraine after age 50.

### Menarche and menopause

Mean age at menarche was 13.2 (SD = 1.4) years for all women and women with MO, and 13.1 years for women with MA. Furthermore, mean age at menopause was 50.1 (SD = 4.3) years for all women, with no difference noted between MO and MA.

More women with MA compared to those with MO reported migraine onset before menarche (11.9 vs. 10.1, *p* < 0.001) and the same age of migraine onset and menarche (7.0 vs. 4.1, *p* < 0.001). Similar proportions were observed between the two groups regarding the cessation of migraine before menopause and the same age at the last migraine attack and menopause. Table 2 summarizes the data.

**Table 2:** Menarche and menopause

	**All women with migraine, ** **No. (%)**	**Migraine without Aura, No. (%)**	**Migraine with Aura, No. (%)**	***P*** **-value**
**Age at Menarche**				
< 10	49 (1.0)			
11	425 (8.9)			
12	956 (20.0)			
13	1357 (28.5)			
14	1162 (24.4)			
15	603 (12.6)			
16	168 (3.5)			
17+	53 (1.1)			
Mean (SD)	13.2 (1.4)	13.2 (1.3)	13.1 (1.4)	0.099*
Missing	52			
**Age at Menopause **				
40–44	448 (10.5)			
45–49	1161 (24.9)			
50–54	2301 (49.3)			
55–59	675 (14.5)			
60+	38 (0.8)			
Mean (SD)	50.1 (4.3)	50.2 (4.3)	50.0 (4.3)	0.329*
Missing	162			
**Migraine Onset**				
Before menarche	433 (10.1)	208 (10.1)	131 (11.9)	
At menarche	215 (5.1)	84 (4.1)	77 (7.0)	
After Menarche	3621 (84.8)	1770 (85.8)	889 (81.1)	< 0.001**
**Migraine Cessation**				
Before menopause	977 (36.0)	445 (33.8)	192 (35.4)	
At menopause	480 (17.7)	237 (18.0)	90 (16.6)	
After Menopause	1258 (46.3)	645 (48.2)	260 (48.0)	0.695**
**Migraine after Menopause**				
Yes	1258 (46.3)	635 (48.2)	260 (48.0)	
No	1457 (53.7)	682 (51.8)	282 (52.0)	0.923**

* = Mann-Whitney U-test

** = Chi-Squared test

### Migraine after menopause

Continued migraine after menopause was reported by 46.3% (*n* = 1258) of all women, with no significant difference between women with MO compared to MA.

**Fig. 5:**
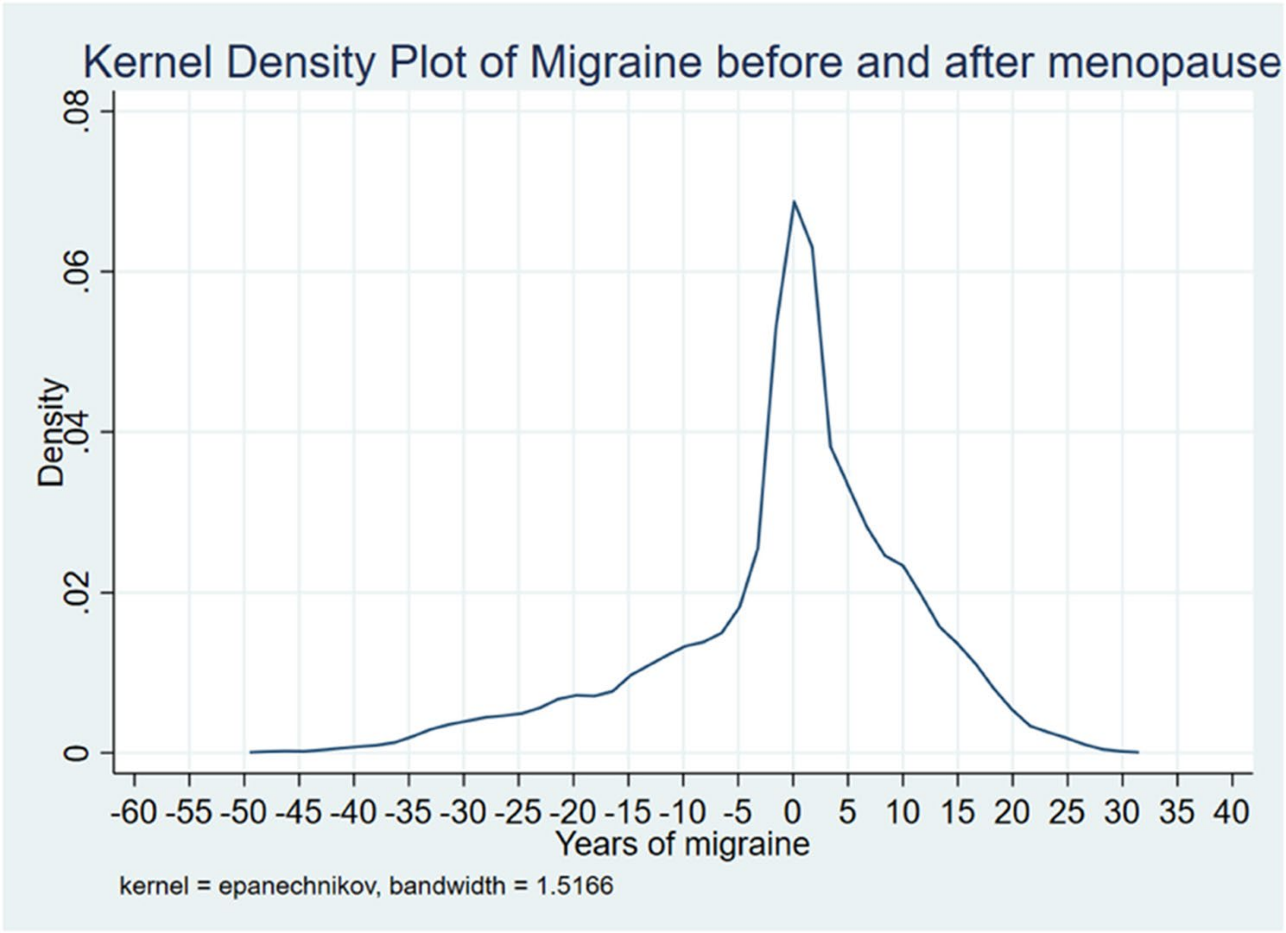
Individual years of migraine before or after menopause. 0= migraine stopped same year as menopause, positive number indicates years of migraine after menopause and negative numbers indicates years of migraine before menopause

Despite this finding, 80.7% of the women reported migraine cessation before 60 years. Table 2; Fig. 5 summarize the data.

Figure 5 shows a kernel density plot displaying individual years of migraine before and after menopause. The plot shows a peak in density around the 0 mark on the x-axis, which represents the time around menopause. This suggests that the occurrence of migraines is most frequent around this period. The density increases sharply as it approaches menopause, indicating a higher frequency of migraines leading up to this time. After menopause, the density decreases, suggesting a reduction in migraine frequency. The x-axis includes negative values, which represent years before menopause. The density is relatively low during these years. The positive values on the x-axis represent years after menopause. The density decreases gradually, suggesting that migraines become less frequent as time progresses post-menopause. Overall, the plot suggests that migraine prevalence peaks around menopause and decreases thereafter.

## Discussion

This large cross-sectional study revealed that migraine cessation usually occurs within the fifth decade of a woman’s life, with an average cessation age of 49.7 years. The average age at menopause was 50.1, implying a temporal relationship between age at last migraine attack and age at menopause. Cessation of migraine before 60 years of age was reported by > 80% of the participants. However, 46.3% of the women continued to experience migraines postmenopause. This finding suggests that menopause, defined as menstruation cessation, serves as a technical marker of time. However, ovarian function is recognized to not abruptly cease following menopause but gradually decline. The data in this study indicate a gradual decline in migraine prevalence with an increase in the duration since the last menstruation. A recent extensive Norwegian study found that, for women born in the 1930 s to the 1960 s, the age at menopause and the duration between menarche and menopause increased by three years [20]. This extension suggests that women experience cyclic hormonal fluctuations related to the menstrual cycle longer, increasing reproductive life expectancy and potentially impacting the prevalence of migraine.

Previous research has documented a decline in migraine prevalence with advancing age [5–7] However, to the best of our knowledge, curves showing migraine cessation in relation to the years since menopause (Fig. 5) have not been previously described, nor have the ages of migraine cessation been illustrated (Fig. 4). Figure 4 shows that many of the women continued to experience migraine postmenopause, followed by a gradual decline. This indicates that menstruation cessation does not necessarily equate to migraine cessation.

A recent review has examined the patterns of postmenopausal migraine, where they discovered inconsistent prevalence, ranging from 14.7 to 61.0% [11], underscoring the requirement for larger-scale research to clarify these aspects, as demonstrated in the present study.

In addition, this study separately investigated the onset and cessation of MO and MA. The onset of migraine in women with MA and MO occurs at approximately the same age, although a trend toward earlier onset in those with MA exists. Our data imply that MA occurs independently of menarche more often than MO, as more women with MA experience migraine before their first menstruation. Furthermore, our findings imply that a slight difference may exist between the groups regarding the co-incidence of menopause and migraine cessation, with a tendency toward fewer women with MA (16.6%) than those with MO (18.0%) reporting this. To the best of our knowledge, this finding has not been previously reported. However, this aligns well with the idea that MA is less closely related to menstruation than MO [9, 10].

Our findings on age of migraine cessation align well with clinical experience, and previous studies on age-related migraine prevalence [1, 5–7]. Even though menopause has been established, migraine continues in a subset of women as noted in one in five women after the age of 60 years in the present study. This insight is critical for managing migraine and information provided to patients, especially regarding symptoms persistence post menopause. Furthermore, it is relevant because of aging demographics, suggesting that migraine prevalence in mid- and late-life women will increase, causing substantial personal and public health difficulties. Migraine management in older adults may be complicated by factors such as polypharmacy and comorbidities [21].

The results of this study indicate the alterations in migraine prevalence during puberty and the menopausal transition, highlighting the significant role of the maturing and aging hypothalamic-pituitary-ovarian axes. With aging, the neuroendocrine system undergoes substantial changes, including fluctuations in estrogen levels when approaching menopause, and attenuation of negative estrogen feedback loops to the pituitary and hypothalamus. Although shifts in female sex hormone levels reflect hypothalamic alterations, they do not directly cause alterations in the prevalence of migraine. The interplay between cerebral changes and hormonal fluctuations during the menopausal transition may critically affect migraine patterns and presentations across reproductive milestones.

MA without headache is more prevalent in older adults and predominantly presents with visual symptoms [7, 22]. However, this impression may be due to the decrease in MO prevalence in women. The prevalence of migraine in men, both MO and MA, may have less age variation but a higher frequency of non-migraine headaches [9]. Notably, MA and MO in the present cohort seemed to follow a similar pattern of cessation. MA in older adults may be clinically challenging because it resembles conditions such as transient ischemic attacks and epileptic seizures [22].

### Strengths and limitations

This study has some limitations. The migraine headache diagnosis and its subtypes were self-reported in a questionnaire rather than confirmed by a physician using the diagnostic gold standard ICHD criteria. However, the questionnaire was derived from the ICHD criteria, which is usually accepted as a valid method for diagnosing migraine, and a similar set of questions based on the ICHD criteria reported sensitivity of 59%, specificity of 99% for lifetime detection of migraine, making it a valid tool for identifying migraine [23].

Moreover, the single question ”Do you have migraine? ‘yes’ or ‘no’ “ has proved to be a valid way of identifying people with migraine [24, 25]; where the “gateway” question in this study is “Have you ever had one or more of the following reoccurring headache conditions– migraine Yes/No”, suggesting that the identification of women with migraine is satisfactory.

In this study, the participants were 34–49 years old at the time of completing the initial questionnaire and 60–74 years old at the time of completing the fourth questionnaire, with migraine questions. Nonetheless, 11.5% (556) of the participants did not report their age at first migraine. This could be due to the differences in perception between childhood and adult migraines, as this may not be recognized in childhood until much later, complicating the determination of migraine onset. Furthermore, 10.8% (332) of the women did not report age at last migraine. Migraine cessation is retrospectively identified based on the time elapsed since the most recent attack. The migraine attack frequency may suddenly or gradually decrease, making it challenging to determine the precise age at which migraines cease. This may have contributed to the missing values. However, few missing values were observed for age at menarche (1.1%, *n* = 52) and age at menopause (3.4%, *n* = 162). Additionally, given that some women were not postmenopausal, missing values existed for age at menopause. A total of 194 women were excluded from the analysis because they reported an age at menopause of ≤ 39 years. Premature menopause is menopause before 40 years and has a prevalence of approximately 1% [26]. We consider it unlikely that premature menopause is more than three times more common among women in the present study. Instead, factors such as medical conditions, medication use, use of hormonal contraception causing amenorrhea or misunderstanding the question, are more probable explanations.

When differentiating between MO and MA in the analysis, women were assigned to either the MO or MA group. Women in the MA group reported MA most of the time. The rationale for this subdivision was the lack of sufficient information about separate migraine attacks to classify MO or MA according to the gold standard set by the ICHD-3. Most patients who experience migraine aura likely do so infrequently, with migraine without aura being more common. Thus, the criterion of ‘Most of the time’ may result in an underestimation. Additionally, since aura can occur independently of headache or during the headache phase, the criterion of ‘before headache’ may also lead to an underestimation. Migraine aura is not uncommon [27]. Conversely, many individuals report nonspecific visual disturbances, such as light sensitivity, which they interpret as migraine aura. In conclusion, the criteria of ‘Most of the time’ and ‘Before headache’ are appropriate, as they provide a conservative estimate. Moreover, the prevalence of MA in NOWAC is similar to what you could expect, based on previous research [27].

Some women experience migraine attacks with and without aura. In our study, we did not facilitate analysis in which one woman could be represented on both the MA or the MO group. Due to the lack of data regarding the frequency of migraine attacks with or without aura, it was more appropriate to categorize the women based on the predominant type of migraine attack they experienced. Furthermore, the absence of data on migraine attack frequency precludes the analysis of the differences in migraine frequency throughout a woman’s life or the identification of life stages with increased migraine impact, such as perimenopause. Existing literature shows a heightened impact of migraine during perimenopause, followed by a reduction post menopause [13]; however, the current study could not explore these patterns because data related to the headache characteristics and frequency was obtained only at one time point in the questionnaire.

The women invited to NOWAC were randomly selected from the national population register. However, it has been found that the responders have a slightly higher level of education and on average have more children compared to the general population of Norwegian women, potentially affecting the generalizability of the results. Nevertheless, the prevalence of migraine found in NOWAC is similar to that found in a recent large-scale review [1]. This study’s examination of the general population, as opposed to earlier research that often focuses on selected groups such as headache clinic patients, provides a broader perspective and strengthens the validity of the findings on migraine in postmenopausal women. Furthermore, the large sample size of 4825 women with migraine strengthened the result’s robustness and statistical power. This study may also be subject to recall bias, as it relies on self-reported data from participants regarding their migraine history and other health-related factors, possibly impacting the study’s findings on migraine onset and cessation. Nonetheless, we do not consider it likely to be systematic differences between the MO group compared to the MA group.

One strength of the present study is the possibility of comparing MO and MA. We found few differences between MO and MA; however, women with MA experienced their first migraine slightly earlier and were more likely to continue having migraine after menopause. This suggests that female sex hormones may play different roles in the two subgroups.

Notably, 9.2% of the women experienced their first migraine attack after 50 years of age, a rarely documented occurrence that warrants further investigation.

Significant gaps remain in understanding migraine cessation, especially regarding the prevalence and impact of postmenopausal migraine. Clinical studies rarely include participants aged > 65 years, though previous reviews suggest that migraine attacks tend to decrease in frequency and intensity with age [11]. Future research is required to confirm these observations, expand our understanding of migraine progression in later life stages, and examine whether the type of menopause (spontaneous or medical/surgical) influences the prevalence and course of migraines following menopause. Future research should explore how migraine attack frequency changes across various life stages and identify potential hormonal or lifestyle predictors of (early) migraine cessation. Such studies could aid in developing personalized treatment strategies.

## Conclusion

Migraine cessation usually occurs during the fifth decade of a woman’s life, and approximately 80% of women in this study reported migraine cessation before 60 years of age. However, the idea that migraines cease following menopause is an oversimplification; nearly half the participants in our study reported migraine post-menopause, and one in five continued to experience them after 60 years of age.

## Supplementary Information

Supplementary Material 1.

Supplementary Material 2.

## Data Availability

No datasets were generated during the current study. The data supporting the findings of this study are available from the corresponding author upon reasonable request.

## Abbreviations

NOWAC: Norwegian Women and Health Study
MO: Migraine without aura
MA: Migraine with aura
ICHD: International Classification of Headache Disorders
SD: Standard deviation

## References

[1] Stovner LJ, Hagen K, Linde M et al (2022) The global prevalence of headache: an update, with analysis of the influences of methodological factors on prevalence estimates. J Headache Pain 23:34 35410119 10.1186/s10194-022-01402-2 PMC9004186

[2] Bugge NS, Grøtta Vetvik KG, Alstadhaug KB et al (2024) Cumulative exposure to Estrogen May increase the risk of migraine in women. Cephalalgia 44. 10.1177/03331024231225972. 10.1177/03331024231225972 38215242

[3] MacGregor EA (2004) Oestrogen and attacks of migraine with and without aura. Lancet Neurol 3:354–361 15157850 10.1016/S1474-4422(04)00768-9

[4] Allais G, Chiarle G, Sinigaglia S et al (2019) Migraine during pregnancy and in the puerperium. Neurol Sci 40:81–88 30880362 10.1007/s10072-019-03792-9

[5] Stewart WF, Wood C, Reed ML et al (2008) Cumulative lifetime migraine incidence in women and men. Cephalalgia 28:1170–1178 18644028 10.1111/j.1468-2982.2008.01666.x

[6] Stewart WF, Lipton RB, Celentano DD et al (1992) Prevalence of migraine headache in the united states. Relation to age, income, race, and other sociodemographic factors. JAMA 267:64–69 1727198

[7] Bigal ME, Liberman JN, Lipton RB (2006) Age-dependent prevalence and clinical features of migraine. Neurology 67:246–251 16864816 10.1212/01.wnl.0000225186.76323.69

[8] MacGregor EA (2020) Menstrual and perimenopausal migraine: A narrative review. Maturitas 142:24–30 33158484 10.1016/j.maturitas.2020.07.005

[9] Chalmer MA, Kogelman LJA, Callesen I et al (2023) Sex differences in clinical characteristics of migraine and its burden: a population-based study. Eur J Neurol 30:1774–1784 36905094 10.1111/ene.15778

[10] Vetvik KG, MacGregor EA (2021) Menstrual migraine: a distinct disorder needing greater recognition. Lancet Neurol 20:304–315 33600767 10.1016/S1474-4422(20)30482-8

[11] Ornello R, Caponnetto V, Frattale I et al (2021) Patterns of migraine in postmenopausal women: A systematic review. Neuropsychiatr Dis Treat 17:859–871 33776441 10.2147/NDT.S285863 PMC7989683

[12] Bushman ET, Varner MW, Digre KB (2018) Headaches through a woman’s life. Obstet Gynecol Surv 73:161–173 29595872 10.1097/OGX.0000000000000540

[13] Ripa P, Ornello R, Degan D et al (2015) Migraine in menopausal women: a systematic review. Int J Womens Health 7:773–782 26316824 10.2147/IJWH.S70073 PMC4548761

[14] Orareme J, Oguga MM, Chinedu P, Maduka et al (2024) Demographic shifts and healthcare: a review of aging populations and systemic challenges. Int J Sci Res Arch 11:383–395

[15] Martins KM, Bordini CA, Bigal ME et al (2007) Migraine in the elderly: A comparison with migraine in young adults. Headache: J Head Face Pain 46:312–316 10.1111/j.1526-4610.2006.00343.x 16492241

[16] De Rijk P, Resseguier N, Donnet A (2017) Headache characteristics and clinical features of elderly migraine patients. Headache: J Head Face Pain 58:525–533 10.1111/head.13247 29235107

[17] Lund E, Dumeaux V, Braaten T et al (2008) Cohort profile: the Norwegian women and Cancer study-NOWAC-Kvinner Og Kreft. Int J Epidemiol 37:36–41 17644530 10.1093/ije/dym137

[18] Lund E, Kumle M, Braaten T et al (2003) External validity in a population-based National prospective study– the Norwegian women and Cancer study (NOWAC). Cancer Causes Control 14:1001–1008 14750540 10.1023/b:caco.0000007982.18311.2e

[19] Headache Classification Committee of the International Headache Society (HIS) (2013) The international classification of headache disorders, edition (beta version). Cephalalgia 33:629–808 23771276 10.1177/0333102413485658

[20] Gottschalk MS, Eskild A, Hofvind S et al (2020) Temporal trends in age at menarche and age at menopause: a population study of 312 656 women in Norway. Hum Reprod 35:464–471 31990353 10.1093/humrep/dez288 PMC7048709

[21] Haan J, Hollander J, Ferrari MD (2007) Migraine in the elderly: a review. Cephalalgia 27:97–106 17257228 10.1111/j.1468-2982.2006.01250.x

[22] Vongvaivanich K, Lertakyamanee P, Silberstein SD et al (2015) Late-life migraine accompaniments: A narrative review. Cephalalgia 35:894–911 25505036 10.1177/0333102414560635

[23] Hagen K, Åsberg NA, Uhlig BL et al (2019) The HUNT4-study: the validity of questionnaire-based diagnosis. J Headache Pain 20:70 31195960 10.1186/s10194-019-1021-0 PMC6734226

[24] Russell MB, Rasmussen BK, Thorvaldsen P, Olesen J (1995) Prevalence and sex-ratio of the subtypes of migraine. Int J Epidemiol 24:612–618 7672904 10.1093/ije/24.3.612

[25] Rasmussen BK, Jensen R, Olesen J (1991) Questionnaire versus clinical interview in the diagnosis of headache. Headache 31:290–295 1860786 10.1111/j.1526-4610.1991.hed3105290.x

[26] Luborsky JL, Meyer P, Sowers MF et al (2003) Premature menopause in a multi-ethnic population study of the menopause transition. Hum Reprod 18:199–206 12525467 10.1093/humrep/deg005

[27] Alstadhaug KB, Hernandez A, Naess H et al (2012) Migraine among Norwegian neurologists. Headache 52:1369–1376 22823901 10.1111/j.1526-4610.2012.02216.x

