# Supplementary material for: Migraine through puberty and menopausal transition—data from the population-based Norwegian Women and Health study (NOWAC)
Source: J Headache Pain. 2025 Jun 20;26(1):145. doi: 10.1186/s10194-025-02083-3 (PMC12180204; doi:10.1186/s10194-025-02083-3)
Supplement: Supplementary file 2 — Supplementary Material 2. [file 10194_2025_2083_MOESM2_ESM.pdf]

Migraine Questions	Answer options
**Have you experienced one or more of the following recurring and/or troublesome headache conditions?**	○ Migraine: Yes/No<br>○ Other kinds of Headache: Yes/No
**If yes, age at start**	Age in years
**If yes migraine, have you had migraine the last year?**	Yes/No
**If No, how old were you when migraine stopped?**	Age in years
**Approximately how many days per month do/did you usually have migraine?**	○ Less than 1 time per month<br>○ 1-6 days/7-14 days<br>○ More than 14 days
**How long does/did the migraine usually last?**	○ Less than 4 hours<br>○ 4 to 72 hours<br>○ More than 72 hours
**How intense is/was the migraine usually?**	○ Mild (does not inhibit daily activity)<br>○ Moderate (inhibiting, but not preventing daily activity)<br>○ Severe (prevents daily activity)
**How often is/was the migraine usually accompanied by and/or characterized by (One or more checks)**<br>• **Pulsating pain**<br>• **Pressing pain**<br>• **Pain on one side of the head (right or left)**<br>• **Pain in the whole head**<br>• **Nausea and/or vomiting**<br>• **Sensitivity to light and/or sound**<br>• **Worsening with physical activity**<br>• **Visual disturbances before the headache (aura)**	○ ☐ Rarely or never/ ☐ Sometimes/ ☐Often<br>○ ☐ Rarely or never/ ☐ Sometimes/ ☐Often<br>○ ☐ Rarely or never/ ☐ Sometimes/ ☐Often<br>○ ☐ Rarely or never/ ☐ Sometimes/ ☐Often<br>○ ☐ Rarely or never/ ☐ Sometimes/ ☐Often<br>○ ☐ Rarely or never/ ☐ Sometimes/ ☐Often<br>○ ☐ Rarely or never/ ☐ Sometimes/ ☐Often<br>○ ☐ Rarely or never/ ☐ Sometimes/ ☐Often

**Do/did you use prescription medication for migraine attacks?**	○ Yes<br>○ No<br>○ No, over-the-counter remedies
**If 'Yes', how often do/did you use it?**	○ Less than once per month<br>○ 1-4 doses per month<br>○ More than 5 doses per month
